# Serous Retinal Detachment in Pre-eclampsia: Case Report and Literature Review

**DOI:** 10.1055/s-0040-1718448

**Published:** 2020-11-30

**Authors:** Joana Teresa Botelho Vasconcelos Raposo, Bruna Carina Da Silva Melo, Nuno Filipe Bernardo Belo Maciel, Sara Dias Leite, Óscar Renato Coutinho Rebelo, Ana Maria Furtado Lima

**Affiliations:** 1 Hospital do Divino Espírito Santo de Ponta Delgada, Ponta Delgada, Portugal

**Keywords:** pre-eclampsia, retinal detachment, pregnancy complications, decreased visual acuity, pré-eclâmpsia, descolamento da retina, complicação da gravidez, diminuição da acuidade visual

## Abstract

Pre-eclampsia (PE) is an obstetric disease with a multifactorial cause that affects ∼ 5% of pregnant women. Vision can be affected with varying severity, and retinal detachment is a very rare complication. It tends to be bilateral, diagnosed postpartum, and more prevalent in women who are primiparous and/or undergo caesarean delivery. The condition typically resolves completely and rarely causes total visual loss in the affected women. Fluorescence angiographic findings support the hypothesis that retinal detachment in PE is secondary to choroidal ischemia from intense arteriolar vasospasm. The present article is related to a case of a 37-year-old pregnant woman who had PE associated with a progressive blurred vision, diagnosed by ophthalmology as serous macular detachment of the retina.

## Resumo

A pré-eclâmpsia (PE) é uma doença obstétrica com uma causa multifactorial que afeta ∼ 5% das grávidas. A visão pode ser afetada com uma gravidade variável, sendo o descolamento de retina uma complicação muito rara. Geralmente é bilateral e seroso, e a sua patogênese encontra-se relacionada com a isquemia coroidal, secundária a um intenso vaso espasmo arteriolar. A maioria dos doentes tem recuperação completa da visão com tratamento conservador. No presente artigo, é relatado um caso de uma grávida de 37 anos que desenvolveu PE com critérios de gravidade associada a um quadro de visão turva progressiva, diagnosticada pela oftalmologia como descolamento macular seroso da retina.

## Introduction

Serous retinal detachment is an unusual cause of visual loss in < 1% of pre-eclampsia (PE) cases. 1 2 It tends to be bilateral, diagnosed postpartum, and more prevalent in women who are primiparous and/or undergo caesarean delivery. 1 The condition typically resolves completely and rarely causes total visual loss in the affected women. 1 3 4 Fluorescence angiographic findings support the hypothesis that retinal detachment in PE is secondary to choroidal ischemia from intense arteriolar vasospasm. 1 3 5

## Case Description

A 37-year-old pregnant woman submitted to in vitro fertilization with no relevant personal medical history and no previous pregnancies. She was admitted at 36 weeks of pregnancy with a diagnosis of PE after presenting high blood pressure (BP) (150/87 mmHg) associated with occasional proteinuria of 300 mg/dL. Blood analysis showed Hb 11.1 g/dL, LDH 355 U/L and AST/TGO 96U/L. During admission, oral nifedipine 10mg was administered to control BP. Despite the improvement of the woman's BP on the second day of hospitalization, there was a slight worsening of blood test results. At that time, induction of labor with misoprostol was started. Due to recurrent variable decelerations and absent baseline Fetal Heart Rate (FHR) variability on cardiotocography (Category III of the American College of Obstetricians and Gynecologists [ACOG] classification), the patient was submitted to an emergent caesarean section (CS). A female was delivered weighing 2,470 g and with an Apgar Score of 10/10.

On the first postoperative day, the patient presented blurred vision and decreased visual acuity and was observed by an ophthalmologist who diagnosed a bilateral serous retinal macular detachment. On the third postoperative day, the patient had a spontaneous recovery in her visual acuity and experienced improvements in her other clinical symptoms, as well as her physical and analytical examinations. The patient was therefore discharged from the hospital medicated with nifedipine and enoxaparin that were started on the first postoperative day as indicated in all patients submitted to an urgent/emergent CS, and was referred to ophthalmology for follow-up.

## Discussion

Pre-eclampsia is an obstetric disease with a complex and multifactorial cause that affects ∼ 5% of pregnant women. 4 6 It usually occurs in the 3 ^rd^ trimester of pregnancy, although it can occur at any time from 20 weeks of gestation to 6 weeks postpartum and is characterized by the presence of hypertension and proteinuria (> 0.3 g in 24-hour urine), thrombocytopenia (platelets < 100,000 3 × 10 ^9^ ), renal insufficiency, impaired liver function or pulmonary edema. 2 6 7 Retinal detachment is a rare complication of PE, affecting 1 to 2% of pregnant women with severity criteria and 10% of eclampsias, and may manifest before or after delivery. 6 The most common ocular finding is severe arteriolar spasm resulting from segmental or generalized constriction of retinal arterioles. Retinal hemorrhage, edema, and “cotton wool spots” may result from arteriolar damage, areas of hypoperfusion or occlusive disease. 3 Serous retinal detachment is an extremely rare complication of hypertensive disease in pregnancy. 1 2 3 6 8 There are few reports in the literature describing it as a cause of vision loss in PE and it involves the separation of the sensorineural retinal from the retinal pigment epithelium as an ophthalmic emergency. Its exact pathophysiology is unknown. 1 3 Doppler evaluation of ophthalmic arterial flow may offer new perspectives regarding the understanding of the pathophysiology, diagnosis and quantification of PE severity. In severe forms, increased impedance of orbital vessels was observed. Studies using fluorescence angiography and green indocyanine angiography indicate that much of the retinal damage presented is caused by changes in the choroid vasculature, including occlusion of choroid and coriocapillary arterioles. 3 After resolution of retinal detachment, there is a change in the shape of irregular focal areas of hyper and hypopigmentation, which correspond to the ischemic stroke – “Elschnig spots”. 1 3 In general, visual acuity gradually improves and visual field defects disappear within 3 months after delivery, with complete recovery of vision. 1 5 6 8 In this particular situation, the timely medical intervention together with a good BP control and adequate patient stabilization allowed a relatively quick reversal of the condition. This case shows the good clinical outcome of conservative therapy applied to retinal detachment in PE.

## Final Considerations

Pregnancies complicated by PE are associated with a worse prognosis for mother and child, hence it is important to be aware of the many complications that may arise. Most patients with retinal detachment in pregnancy-induced hypertension have full spontaneous resolution within a few weeks without any long-term complications. Medical treatment with antihypertensive drugs and steroids may be helpful.
